# Oxidized Low-Density Lipoprotein (Ox-LDL) and Triggering Receptor-Expressed Myeloid Cell (TREM-1) Levels Are Associated with Cardiometabolic Risk in Nonobese, Clinically Healthy, and Young Adults

**DOI:** 10.1155/2019/7306867

**Published:** 2019-02-28

**Authors:** Cecilia Maria Passos Vázquez, Jamille Oliveira Costa, Lays Gisele Santos Bomfim, Liliane Viana Pires, Danielle Góes da Silva, Kiyoshi Ferreira Fukutani, Amélia Ribeiro de Jesus, Natanael de Jesus Silva, Gleiciane de Jesus Santana, Tatiana Rodrigues de Moura, Kiriaque Barbosa

**Affiliations:** ^1^Federal University of Sergipe (UFS), Aracaju, SE, Brazil; ^2^São Paulo University (USP), Ribeirão Preto, SP, Brazil; ^3^Federal University of Bahia (UFBA), Salvador, BA, Brazil

## Abstract

Oxidative and inflammatory substances play an important role in the genesis of processes related to cardiometabolic risk. High levels of oxidized low-density lipoprotein (Ox-LDL) and of triggering receptor-expressed myeloid cells (TREM-1) are associated with cardiovascular and inflammatory diseases. In this study, we evaluate the association of the plasma concentrations of Ox-LDL and serum levels of circulating TREM-1 (sTREM-1) with the components of cardiometabolic risk (CMR) and other associated risk parameters. Although the individuals in this study were young, nonobese, and did not have signs, symptoms, and diagnosis of diseases, they already presented components of CMR. Ox-LDL lipid fraction correlated positively with CMR-related markers: body mass index (BMI), waist circumference (WC), body fat percentage, total cholesterol, LDL-c, VLDL-c, triglycerides, atherogenic cholesterol, and atherogenic index. Among these parameters, atherogenic cholesterol had a greater predictive effect for Ox-LDL alterations. Individuals with higher serum concentrations of sTREM-1 presented higher values for BMI, WC, triglycerides, VLDL-c, and atherogenic cholesterol. WC showed an effect on the association between the sTREM-1's inflammatory response and the components of CMR. The association of oxidative and inflammatory markers with anthropometric parameters and atherogenic cholesterol in nonobese, clinically healthy, and young individuals suggests the importance of early evaluation of these markers in order to prevent future cardiac events.

## 1. Introduction

The term cardiometabolic risk (CMR) encompasses the risk components that contribute to the development of cardiovascular disease (CVD) and type 2 diabetes. Oxidative stress and inflammatory response are associated with components of CMR, such as systemic arterial hypertension, hyperglycemia, altered lipid profile (low HDL-c and high triglyceride levels), and abdominal obesity, in addition to associated risk factors, such as dyslipidemia (high LDL-c, VLDL-c, total cholesterol, atherogenic index, and atherogenic cholesterol) and insulin resistance (elevated HOMA-IR–Homeostatic Model Assessment of Insulin Resistance) [1–6] . The associations of oxidative stress and inflammation with the components of CMR have been evidenced in the context of the intense production of reactive oxygen species (ROS) and inflammatory interleukins related to metabolic alterations, such as increased insulin resistance and visceral adiposity [4, 6, 7].

The oxidative stress related to metabolic alterations is an early damage and is strongly associated with CMR and noncommunicable diseases (NCDs), even in healthy and young individuals [6, 8–13]. ROS produced from oxidative stress induce oxidation of LDL-c lipid fraction, generating oxidized LDL lipid fraction (Ox-LDL). Ox-LDL is one of the biomarkers of oxidative stress and is recognized as a strong predictor of CVD, since it has a positive association with central obesity, CMR markers, and atherosclerosis [6, 8–12].

The cell surface receptor TREM-1 (triggering receptor-expressed myeloid cells) is a glycoprotein expressed in neutrophils, subsets of monocytes, and macrophages. This receptor not only plays a role in the innate immune response but also in inflammatory vascular responses [14]. Recently, it has been reported in the literature that the soluble form of TREM-1, known as sTREM-1, is increased in patients with coronary artery disease, diabetes, and obesity, suggesting sTREM-1 as a potential biomarker that reflects carotid plaque instability and chronic inflammation associated with obesity and insulin resistance [15, 16].

Clinically healthy, nonobese, and young individuals already have risk factors associated with oxidative stress and inflammation. Therefore, measuring markers that predict oxidative stress and inflammation is a reliable strategy to diagnose early onset of CMR. However, measuring markers of oxidative stress and inflammation in clinical practice is very expensive. Thus, this study is aimed at evaluating the association of oxidative stress (Ox-LDL) and inflammation (sTREM-1) markers with anthropometric, clinical and biochemical parameters, including the components of CMR.

## 2. Methods

### 2.1. Study and Sampling Design

This is a cross-sectional study with a convenient sampling selection. The sample consisted of young adults of both sexes, aged between 18 and 25 years old, and students of the health area from public and private universities in the state of Sergipe, Brazil. We calculated the sample size according to Miot's methodology [17], using the prevalence of 9.9% of high waist circumference in college students [18], significance level of 5%, test power of 80%, and population size of 8,951, considering the number of students enrolled in health courses. The minimum sample size of 135 individuals was estimated. The final sample consisted of 187 individuals.

### 2.2. Exclusion Criteria

As exclusion criteria, we considered the evidence of diseases related to oxidative stress, chronic inflammation, and hydroelectrolytic imbalances; changes in body composition in the last six months or changes in nutrient absorption and/or metabolism; use of medications or nutritional treatment that changed energy balance, food consumption, lipid profile, plasma insulin concentrations, and glucose metabolism; regular contraceptive use until at least two months prior to the participation on this study; unstable weight in the last six months (allowing fluctuation of up to 10% of body weight); adoption of specific diets in at least three months; pregnant and lactating women; and elite athletes.

### 2.3. Analysis of Biological Samples

We performed blood collection by venipuncture after 12 hours of fasting and without alcohol, coffee, or tea intake for 24 hours. The heparin and plasma samples were separated by centrifugation at 2465 g at 5°C for 15 minutes and stored at -80°C. Serum concentrations (mg/dL) of glucose, total cholesterol, high-density lipoprotein (HDL-c), and triglycerides were analyzed by colorimetric or turbidimetric method. The serum concentration (IU/mL) of insulin was analyzed by electrochemiluminescence. HOMA-IR value was calculated from the fasting insulin and glucose dosages. Plasma concentrations of Ox-LDL and serum levels of the inflammatory molecule sTREM-1 were analyzed by enzyme-linked immunosorbent assay (ELISA), using the specific analysis kit (Mercodia, Uppsala, Sweden) and DuoSet-Human TREM-1 (R&D Systems, Minneapolis, USA).

### 2.4. Anthropometric and Body Composition Assessment

Height was measured to the nearest 1 mm using a stadiometer (Altura Exata, Minas Gerais, Brazil) and body weight to the nearest 100 grams using a digital scale (Líder, P 180M, São Paulo, Brazil). We calculated body mass index (BMI) by dividing the weight (kg) by the squared height (m^2^) and classified according to the cutoff points proposed by the World Health Organization [19]. To measure waist circumference, we adopted the midpoint between the last rib and the iliac crest, using an inelastic flexible tape [19].

We performed a horizontal tetrapolar bioelectrical impedance analysis (BIA) (Biodynamics 310 model, Washington, USA), with fasting of at least four hours and no physical exercise for at least eight hours before the examination. The classification of body fat percentage followed the cutoff points for healthy men and women proposed by Lohman et al. [20].

### 2.5. CMR Components

We diagnosed CMR components according to the International Diabetes Federation criteria [1]: abdominal obesity (waist circumference ≥80 cm for women and ≥90 cm for men), fasting hyperglycemia (≥100 mg/dL), hypertriglyceridemia (≥150 mg/dL), low HDL-c (<50 mg/dL for women and <40 mg/dL for men), and high blood pressure (systolic pressure ≥130 mmHg and diastolic pressure ≥85 mmHg). Systolic and diastolic blood pressures were measured using a mercury sphygmomanometer with an accuracy of 2 mmHg, according to Perloff et al. [21].

### 2.6. Statistical Analysis

**Figure 1:**
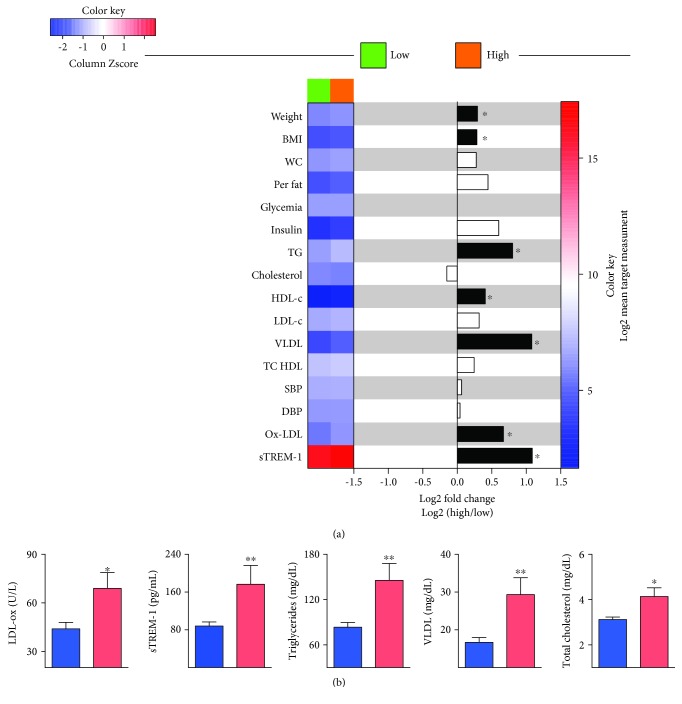
Markers of oxidative stress (Ox-LDL) and inflammation (sTREM-1) and other variables of interest related to the components of the CMR among individuals with waist circumference above and below the recommendation. (a) The heat map was assessed by unsupervised bidirectional hierarchical cluster analysis (Ward's method) and normalized by Zscore of individuals categorized by waist circumference above or below the profile recommendation. Differences in marker levels between above compared to below by *t* of the Student *t*-test was considered significant with a *p* value < 0.05 and statistically significant differences were highlighted in black. Fold changes were calculated and statistically significant differences are highlighted in black. (b) Biochemistry and inflammatory parameters that displayed statistically significant differences between the waist circumference tested by Mann–Whitney *U* test. BMI: body mass index; WC: waist circumference; Per fat: percentage fat; TG: triacylglycerol; HDL-c: high-density lipoprotein; LDL-c: low-density lipoprotein; VLDL-c: very low-density lipoprotein; DBP: diastolic blood pressure; SPB: systolic blood pressure; TC HDL: atherogenic cholesterol; Ox-LDL: oxidized low-density lipoprotein; sTREM-1: soluble triggering receptor-expressed myeloid cells-1.

After verifying the data distribution using the Kolmogorov–Smirnov test, we performed the Spearman test to verify the correlation of the oxidative stress (Ox-LDL) and inflammation (sTREM-1) markers with other variables of interest related to the CMR components (Table 1). The Mann–Whitney *U* test was adopted for comparison between the groups categorized by the risk marked by waist circumference (Figure 1). We performed multivariate linear regression with Ox-LDL values as the dependent variable. The model was adjusted for sex and age. 95% confidence interval was used to describe the values of the linear regression coefficient (*β*) (Table 2). The level of statistical significance was set at 5% of probability (*p* < 0.05).

An unsupervised bidirectional hierarchical cluster analysis (Ward's method) with a 100X bootstrap was used to test whether individuals categorized according to waist circumference could be grouped separately based on the general expression of the oxidative stress (Ox-LDL) and inflammation (sTREM-1) markers and other variables of interest related to the CRM components (Figure 1).

### 2.7. Ethical Aspects

The study was approved by the Human Research Ethics Committee of the Federal University of Sergipe (C.A.A.E.: 0113.0.107.000-11). In accordance with the principles of the Declaration of Helsinki, all volunteers were informed about the study protocol and then signed the consent form. The volunteers were informed about the methods and procedures used in the data collection, the possible benefits and inconveniences, the privacy of results, and the voluntariness of participation.

## 3. Results

### 3.1. Characterization of the Study Population

We evaluated 187 young adults, with a mean age of 21 ± 2.0 years old and predominance of females (74.5%). Although the individuals in this study were young, nonobese, and clinically healthy (absence of signs, symptoms, and diagnosis), they already had CMR components. The CMR components with the highest frequencies in this population were low HDL-c, hypertriglyceridemia, and abdominal obesity (Table 3).

Regarding other biochemical parameters, almost 20% presented hypercholesterolemia, followed by changes in atherogenic cholesterol, HOMA-IR indicator, and elevated body fat (data not shown in table).

### 3.2. Markers of Oxidative Stress and Inflammation

The markers of oxidative stress (Ox-LDL) and inflammation (sTREM-1) were correlated with anthropometric and biochemical variables related to the components of CMR (Table 1).

After adjustment for waist circumference, Ox-LDL remained positively correlated with biochemical markers related to the CMR components: triglycerides, total cholesterol, LDL-c, VLDL-c, atherogenic cholesterol, and atherogenic index. sTREM-1 no longer correlated with the CMR components after adjusting for wasting circumference (Table 1).  Figure 1(a) corroborates the effect of waist circumference on the association of the markers of oxidative stress and inflammation with the CMR components.

### 3.3. Determinants of Ox-LDL Dosages

In the multivariate linear regression model (Table 2), we verified that atherogenic cholesterol was the risk parameter with the greatest prediction effect for Ox-LDL dosages. The increase of 1 mg/dL in the serum levels of atherogenic cholesterol was able to predict an increase of 0.553 U/L in Ox-LDL, regardless of the effect of age, sex, presence of at least one component of CMR, and triglycerides. The presence of at least one component of CMR and triglycerides also had a predictive effect on Ox-LDL dosages, but with lower intensity than atherogenic cholesterol.

### 3.4. Comparison of Groups Categorized by Waist Circumference

According to the hierarchical clustering analysis (Figure 1(a)), categorized by the waist circumference values, a higher expression of the anthropometric and biochemical parameters related to CMR was observed among individuals with elevated waist circumference. Ox-LDL, sTREM-1, triglycerides, VLDL-c, and total cholesterol were the most expressed parameters in individuals with elevated waist circumference (Figure 1(b)).

## 4. Discussion

Our study was the first to report that serum sTREM-1 presented a positive and significant correlation with waist circumference and other several indicators related to the CMR. This association can be justified by the inflammatory process in visceral adipose tissue [15].

Studies show that excessive abdominal fat is the risk factor more strongly associated with the CMR [22–28]. Body fat, especially visceral fat, reflected by waist circumference, has a strong association with chronic inflammation [14, 29–32]. This inflammation occurs due to a greater secretion of inflammatory cytokines (TNF-*α*, IL-1*β*, and IL-6) and lower secretion of the anti-inflammatory cytokine IL-10 by the visceral adipocytes. The imbalance between these cytokines impacts several body functions, such as food intake control, energy balance, immune system, angiogenesis, blood pressure, lipid metabolism, and body homeostasis, strongly correlated with cardiovascular diseases. In addition, adipokines secreted by visceral adipose tissue, such as leptin, adiponectin, and TNF-*α*, play a key role in the tissue sensitivity to insulin, favoring the onset of insulin resistance and tissue inflammation. [22–28].

Recently, a study suggested that the increased expression of TREM-1 in tissue biopsies and circulating neutrophil and monocytes may precede changes in serum levels of other inflammatory markers in individuals with diabetes and with TREM-1 presenting a possible role in the underlying pathophysiology of obesity and associated comorbidities [15]. In addition, higher concentrations of sTREM-1 levels have already been observed in adults and obese children, suggesting that this marker may be associated with cardiovascular risk [16].

Obesity markers, such as BMI, cutaneous folds, and waist circumference, reflect the development of inflammatory processes and cardiometabolic risk. A study conducted with clinically healthy, young adults found good identification of cardiometabolic markers through the evaluation of body fat percentage and BMI [27], which are simpler and least costly parameters. Therefore, anthropometric and body composition markers are satisfactory to screen metabolic abnormalities related to overweight and consequently to inflammation [27, 33].

As sTREM-1 has been suggested to be involved in the onset of acute and chronic inflammation [15], the oxidation of LDL-c lipid fraction has an important etiological role in the atherogenic process and metabolic alterations that follow the insulin resistance and the CMR components [34–36]. In our study, Ox-LDL dosages showed a positive and significant correlation with several biochemical indicators related to CMR, suggesting the beginning of the inflammatory process, oxidative stress, and a greater predisposition for cardiovascular diseases in even nonobese, clinically healthy, and young individuals.

Among the biochemical markers correlated with Ox-LDL, the identification of atherogenic cholesterol as predictor for Ox-LDL alteration has greater clinical importance. In addition to being a good predictor for CVD, the use of atherogenic cholesterol in clinical practice has a number of advantages in relation to the isolated use of other lipoproteins. First, atherogenic cholesterol has more atherogenic lipoproteins, such as VLDL-c, IDL-c, and LDL-c [37]. Second, its calculation is easily quantified by subtracting the values of total cholesterol and HDL-c (non-HDL cholesterol = TC–HDL − c) [38]. Third, its value is not affected by hypertriglyceridemia, not requiring prolonged fasting [39].

Simple, routine, and less expensive measures, such as the serum atherogenic cholesterol dosage, can predict the plasma concentration of Ox-LDL about 50%, which is considered an expensive marker, but which early evaluates the oxidative stress and plays an important role in the atherogenic process [34–36, 40]. This finding becomes an important tool in clinical practice, since it makes possible the early evaluation of clinically healthy, young adults, in order to prevent the development of CVD.

Our results showed an inflamed and atherogenic profile in the young adults that may be related to sedentary lifestyle and inadequate eating habits. Unhealthy lifestyles are very common among college students, since the academic life is characterized by stress, activity overload, consumption of foods with higher energy density and low levels of micronutrients and fibers, meal skipping, and lack of time for exercise. In turn, these environments and behaviors contribute to the development of important metabolic alterations related to diabetes, hypertension, and dyslipidemia, which are considered the main causes of morbidity and mortality in adults [41–46].

College students from low- and middle-income countries are more exposed to eating habits related to the increase in CMR, due especially to the excessive consumption of processed and ultraprocessed foods. The consumption of these foods has been more frequent in the university population due to their practicality, hyperpalatability, low cost, and media appealing. The unfavorable nutritional composition and the high consumption of processed and ultraprocessed foods make them potential risk factors for abdominal obesity, hypertriglyceridemia, reduction of HDL-c, and other NCDs. Given this context, this subject has been the target of several studies [47–53].

Encouraging the adoption of appropriate lifestyle, such as healthy eating habits and exercise practice, contributes to the prevention of CVD. Tracking alterations, consequence of inadequate habits, using less costly monitoring markers in clinical practice, are more achievable for early intervention of unfavorable clinical outcomes.

## 5. Conclusion

The results of the study showed that Ox-LDL and sTREM-1 were associated with biochemical and anthropometric markers related to CMR. In addition, atherogenic cholesterol, which is considered a simple and less expensive measure in clinical practice and recognized as a strong predictor of CVD, was able to predict an increase in Ox-LDL concentration.

It is known that dosing markers of oxidative stress and inflammation in clinical practice is expensive and has a complicated logistics. Therefore, we suggest that the evaluation of atherogenic cholesterol should be part of the process of prevention and control of CVD and inflammatory processes related to CMR. Thus, in addition to predicting future cardiac events, the suggested evaluation is viable, practical, and inexpensive in clinical practice, and can be performed by any trained health professional.

## Figures and Tables

**Table 1:** Correlation profile between oxidative stress markers (Ox-LDL), inflammation (sTREM-1), and other variables of interest related to the cardiometabolic risk components.

Variables	Ox-LDL		sTREM-1	
	Gross	With adjustment for WC	Gross	With adjustment for WC
Age				
*r*	0.113	-0.020	0.074	0.045
*p*	0.17	0.89	0.58	0.75
Weight				
*r*	0.155	0.067	0.164	-0.166
*p*	0.05	0.64	0.22	0.24
BMI				
*r*	0.202	0.105	0.332	0.081
*p*	^∗∗^0.01	0.46	^∗∗^0.01	0.57
WC				
*r*	0.202	0.142	0.393	0.155
*p*	^∗∗^0.01	0.32	^∗∗^≤0.01	0.27
Per fat				
*r*	0.172	0.101	0.255	0.174
*p*	^∗^0.03	0.48	0.05	0.21
Glycemia				
*r*	-0.040	0.101	-0.236	-0.179
*p*	0.63	0.48	0.08	0.20
Insulin				
*r*	0.110	0.092	0.068	0.040
*p*	0.22	0.52	0.62	0.78
HOMA-IR				
*r*	0.101	0.092	0.022	0.022
*p*	0.25	0.52	0.87	0.87
Triacylglycerol				
*r*	0.497	0.469	0.346	0.193
*p*	^∗∗^≤0.01	^∗∗^≤0.01	^∗∗^≤0.01	0.17
Cholesterol				
*r*	0.591	0.649	0.205	0.139
*p*	^∗∗^≤0.01	^∗∗^≤0.01	0.12	0.32
HDL-c				
*r*	-0.062	-0.020	-0.010	0.003
*p*	0.45	0.89	0.94	0.98
LDL-c				
*r*	0.617	0.680	0.210	0.123
*p*	^∗∗^≤0.01	^∗∗^≤0.01	0.11	0.38
VLDL-c				
*r*	0.505	0.473	0.344	0.189
*p*	^∗∗^0.00	^∗∗^0.00	^∗∗^0.00	0.18
TC HDL				
*r*	0.666	0.733	0.263	0.155
*p*	^∗∗^≤0.01	^∗∗^≤0.01	^∗^0.04	0.27
Aterogenic Index				
*r*	0.566	0.696	0.257	0.124
*p*	^∗∗^≤0.01	^∗∗^≤0.01	0.05	0.38
SBP				
*r*	-0.047	-0.059	-0.063	-0.184
*p*	0.57	0.68	0.64	0.19
DBP				
*r*	0.098	0.080	-0.253	-0.323
*p*	0.23	0.58	0.06	^∗∗^≤0.01

*r*, Spearman correlation coefficient; ^∗^*p* < 0.05, ^∗∗^*p* ≤ 0.01; BMI: body mass index; WC: waist circumference; Per fat: percentage fat; HDL-c: high-density lipoprotein; LDL-c: low-density lipoprotein; VLDL-c: very low-density lipoprotein; TC HDL: atherogenic cholesterol; SPB: systolic blood pressure; DBP: diastolic blood pressure.

**Table 2:** Multivariate linear regression analysis of oxidized low-density lipoprotein (Ox-LDL) dosages in clinically healthy, young adults.

Dependent variable	Independent variables	*β* (CI 95%)	*p*	*r* ^2^	*p*
Ox-LDL	Sex^1^	-0.07 (-10.86, 2.99)	0.26	0.503	<0.001
	Age	0.06 (-0.53, 1.82)	0.28		
	Presence of CMR^2^	0.17 (1.34, 14.57)	0.01		
	Atherogenic cholesterol^3^	0.55 (0.26, 0.42)	0.00		
	Triacylglycerol	0.19 (0.02, 0.17)	0.00		

^1^Categories: 0, female and 1, male. ^2^Categories (presence of at least 1 component of the CMR): 0, no risk and 1, risk. ^3^Total cholesterol–HDL-c. CMR- cardiometabolic risk components.

**Table 3:** Demographic data and frequency of cardiometabolic risk components (CMR) in young, nonobese, and clinically healthy subjects (*n* = 187).

	*n* (%) or *X* ± SD
*Demographic data*	
Sex (female)	140 (74.5)
Age (years)	21, 53 ± 2.0
Weight (kg)	58.2 ± 9.8
Height (cm)	164.8 ± 7.9
Body mass index (kg/m^2^)	21.7 ± 2.8
*Components of CMR*	
Abdominal obesity^a^	16 (8.5)
Hyperglycemia^b^	9 (5.0)
Hypertriglyceridemia^c^	14 (7.7)
Low HDL-c^c^	37 (20.3)
High blood pressure^d^	2 (1.0)

Data presented in absolute (*n*) and relative (%) frequencies or mean (x) and standard deviation (SD); ^a^*n* = 169; ^b^*n* = 181; ^c^*n* = 182; ^d^*n* = 165.

## Data Availability

The descriptive data on sociodemographic characteristics and the anthropometric, clinical, and biochemical parameters used to support the results of this study are available from the corresponding author, upon request, by kiribarra@yahoo.com.br.
